# m^6^A-Mediated Upregulation of LINC00857 Promotes Pancreatic Cancer Tumorigenesis by Regulating the miR-150-5p/E2F3 Axis

**DOI:** 10.3389/fonc.2021.629947

**Published:** 2021-02-18

**Authors:** Xiangrui Meng, Yanyao Deng, Shuhan He, Li Niu, Hongwei Zhu

**Affiliations:** ^1^ Department of Hepatopancreatobiliary Surgery, The Third Xiangya Hospital, Central South University, Changsha, China; ^2^ Department of Gastroenterology, The Third Xiangya Hospital, Central South University, Changsha, China; ^3^ Department of Neurology, The First Hospital of Changsha, Changsha, China

**Keywords:** M^6^A, LINC00857, MiR-150-5p, E2F3, pancreatic cancer

## Abstract

The mortality and morbidity rates of pancreatic cancer (PC) have been increasing over the past two decades. Recent evidence indicates that long non-coding RNAs (lncRNAs) are usually dysregulated in the tumorigenesis and progression of PC. In the present study, we showed that the expression of LINC00857 was upregulated in PC and associated with poor prognosis based on the Gene Expression Profiling Interactive Analysis (GEPIA) database and validated in our PC tissues and cell lines. N^6^-Methyladenosine (m^6^A) was highly enriched within LINC00857 and enhanced its RNA stability. Knockdown of LINC00857 remarkably inhibited the proliferation and promoted the apoptosis of PC cells. Then, by using bioinformation analysis and verified experiments, we identified that LINC00857 functioned as a competing endogenous RNA (ceRNA) for sponging miR-150-5p, leading to the upregulation of its target E2F3 in PC cells. Taken above, our study revealed a potential ceRNA regulatory pathway in which LINC00857 modulates E2F3 expression by binding to miR-150-5p, ultimately promoting tumorigenesis in PC. LINC00857/miR-150-5p/E2F3 regulatory axis may be taken as an alternative therapeutic target for treating PC.

## Introduction

As a life-threatening malignant tumor around the world, pancreatic cancer (PC) is difficult to diagnose and treat (1). This is mainly attributed to the late diagnosis (2). Surgical resection is the only curative treatment but many patients lose the opportunity due to being diagnosed at the advanced stage (3). In addition, its clinical features are short duration, rapid development and deterioration (4). Despite the progress in treatment, post-operative recurrence and metastasis still hinder the improvement of 5-year overall survival for PC patients (5). Thus, the new strategy is needed to be explored for treating PC.

Long non-coding RNAs (lncRNAs) are a variety of RNA molecules consisting of more than 200 nucleotides without protein-coding potential (6). Increasing evidence shows that lncRNAs play a crucial role in regulating gene expression on both transcriptional and post-transcriptional levels (7, 8), which is vital to maintaining the normal biological functions (9–11). It has also been widely reported that lncRNAs are suitable as promising biomarkers for PC (12–16). Thus, further insight into the lncRNA-dependent gene-regulatory mechanisms will provide useful prognostic biomarkers and individual treatments for PC. Recently, LINC00857 was suggested to be closely associated with the tumorigenesis and progression of various cancers. It was first found to be upregulated in lung cancer patients and could facilitate the proliferation and invasion of lung cancer cells (17). Although there are already studies on LINC00857 in other cancers, the physiological function of LINC00857 in the progression of PC is still largely unclear.

Based on the Gene Expression Profiling Interactive Analysis (GEPIA) database, we found that high expression of LINC00857 was associated with poor prognosis in PC. Our study further validated that LINC00857 was upregulated in PC tissues and cells. The m^6^A mark improved the stability of methylated LINC00857 transcripts by decreasing the RNA degradation rate, which may partially account for the upregulation of LINC00857 in PC. Functional experiments showed that LINC00857 promoted the proliferation and inhibited the apoptosis of PC cells. LINC00857 sponges miR-150-5p as a competing endogenous RNA (ceRNA) to increase E2F3 expression. This study elucidates the clinical significance and regulatory mechanism of LINC00857 in PC and provides a potential therapeutic target for PC patients.

## Materials and Methods

### GEPIA Database

The GEPIA (http://GEPIA.cancer-pku.cn/index.html) online database was performed to analyze gene sequencing data from the cancer genome atlas (TCGA) and Genotype-Tissue Expression (GTEx). GEPIA database analysis was used to assess the expression levels of LINC00857 or E2F3 and find the association between the survival rate and LINC00857 or E2F3 expression in PC patients.

### Clinical Samples

Twenty pairs of PC tissues and adjacent normal tissues were obtained from patients in the Third Xiangya Hospital of Central South University from January 2017 to December 2019. The patients were diagnosed pathologically abiding by the WHO classification system by at least two advanced clinical pathologists and treated neither by chemotherapy nor radiotherapy prior to surgical resection. This project received approval from the hospital. All patients have signed written informed consent and the study was conducted in line with the Declaration of Helsinki. All samples were stored in liquid nitrogen after resection and before use.

### Cell Culture

The human PC cell lines Panc1, CFPAC-1, BXPC3, CAPAN2, and SW1990, as well as human pancreatic ductal epithelial (HPDE) cells were provided by ATCC. All cells were incubated in DMEM (Hyclone) added with 10% fetal bovine serum (FBS) (Hyclone) and without antibiotics. Cells received the incubating process in wet atmosphere containing 5% CO_2_ at 37°C.

### RNA Extraction and qRT-PCR

TRIzol (Invitrogen) was used to extract total RNAs from cells and tissues. The PARIS™ Kit (Invitrogen) was applied to isolate RNAs from nucleus and cytoplasmic fractions. With regard to lncRNA and mRNA, a PrimeScript™ RT reagent Kit (TaKaRa) was applied to obtain cDNA. The TB Green™ Premix Ex Taq™ II (TaKaRa) was employed to conduct real-time PCR. GAPDH was adopted as the internal control. As for miRNA, a miRcute Plus miRNA First-Strand cDNA Kit (TIANGEN) was applied to the generation of cDNA. A miRcute Plus miRNA qPCR Kit (SYBR Green) was employed to conduct real-time PCR. U6 small nuclear RNA was applied as the internal control. The ABI 7500 real-time PCR system (Applied Biosystems) was applied to perform real-time PCR. The 2^−ΔΔCt^ method was adopted for calculating the relative expression of RNA. Primers were synthesized by Sangon Biotech (Shanghai, China). Primer sequence is detailed below: LINC00857, forward: 5′-CCCCTGCTTCATTGTTTCCC-3′ and reverse: 5′-AGCTTGTCCTTCTTGGGTACT-3′; E2F3, forward: 5′-TGACCCAATGGTAGGCACAT-3′ and reverse: 5′-CATCTAGGACCACACCGACA-3′; GAPDH, forward: 5′-GTCTCCTCTGACTTCAACAGCG-3′ and reverse: 5′-ACCACCCTGTTGCTGTAGCCAA-3′. miR-150-5p, forward: 5′- ACACTCCAGCTGGGTCTCCCAACCCTTGTA-3′ and reverse: 5′-CTCAACTGGTGTCGTGGAGTCGGCAATTCAGTTGAGCACTGGTA-3′; U6 snRNA forward: 5′-CTCGCTTCGGCAGCACA-3′, reverse: 5′-AACGCTTCACGAATTTGCGT-3′.

### m^6^A RNA Methylation Quantification

m^6^A RNA Methylation Quantification Kit (Abcam) was used to assess total m^6^A levels of the extracted RNA. The RNA high binding solution was used for the binding of total RNA and control RNA to the wells. Specific antibodies were used to capture and detect m^6^A. Then, we examined the absorbance at 450 nm for assessing the m^6^A signal after adding enhancer and color developing solutions.

### RNA Immunoprecipitation

A Magna RNA-Binding Protein Immunoprecipitation Kit (Millipore) was employed to conduct RNA immunoprecipitation. Briefly, the cells were obtained to carry out RNA immunoprecipitation (RIP) experiments using an m^6^A antibody (Synaptic Systems) or Ago2 antibody (Abcam). IgG was adopted as negative control. As mentioned previously, qRT-PCR was applied to detect the RNAs after its co-precipitation.

### Cell Transfection

The small short hair RNA (shRNA) targeting METTL3 (sh-METTL3) and its negative control (sh-NC), LINC00857 small interfering RNA (si-LINC00857) and its negative control (si-NC), E2F3 overexpression plasmid (pc-E2F3) and its negative control (pc-NC) were provided by RiboBio (Guangzhou, China). GenePharma Company (Shanghai, China) contributed to synthesizing miR-150-5p mimic and inhibitor or their negative controls (miR-NC and anti-miR-NC). LipofectamineTM3000 transfection reagent (Invitrogen) was employed for building cells transfecting process.

### CCK-8

For ascertaining the proliferation of cells, cell viability was determined using CCK-8 reagent (Dojindo). Cells underwent the seeding process into 96-well plates. After the addition of CCK-8 reagent into respective wells at the specified time point, it was incubated in the incubator containing 5% CO_2_ for 2 h, with the temperature set to 37°C. Then, the absorbance was examined at 490 nm for assessing cell proliferation.

### Colony Formation Assay

We seeded cells undergone transfection in six-well plates with DMEM containing 10% FBS and incubated throughout the night. Fourteen days later, methanol was adopted for fixing cells prior to the use of 0.1% crystal violet to stain treatment. Under a light microscope, we counted the colonies.

### Flow Cytometry

Cells were gained after transfection for 48 h. Then, the Annexin V-fluorescein isothiocyanate (FITC)/propidium iodide (PI) Apoptosis Detection Kit (Yeasen) was employed to stain the cells. Flow cytometer (Thermo Fisher Scientific) was employed for gaining fluorescence signals and ascertaining the apoptosis rate.

### Dual-Luciferase Reporter Assay

The putative miR-150-5p binding sites were assessed in LINC00857/E2F3 3′-UTR. The pMIR-REPORT™ (RiboBio), covering wild type (WT), or mutant (MT) LINC00857/E2F3 3′-UTR sequences was employed for carrying out the dual-luciferase reporter assay. Accompanied by WT or MT LINC00857/E2F3 3′-UTR vector, cells (1×10^5^) saw transient co-transfection with the miR-150-5p mimic or the negative control. Two days later, the cells were obtained. The Luc-Pair™ Duo-Luciferase Assay Kit (Yeasen) was used to determine the luciferase results.

### Western Blot Analysis

BCA Protein Assay Kit (Sigma) was applied to extracting the total proteins derived from PC cells and detect protein concentration. Proteins underwent the fractionation with 10% sodium dodecyl sulfate/polyacrylamide gel electrophoresis (SDS/PAGE). Then we placed the protein into the polyvinylidene fluoride (PVDF) membrane after separation. The membranes underwent incubation with METTL3 antibody (Abcam), E2F3 antibody (Santa Cruz Biotechnology), and β-actin (Santa Cruz Biotechnology) at 4°C overnight. Subsequently, blotted membranes underwent 2h of incubation treatment by HRP-conjugated secondary antibody at ambient temperature. ECL Substrates (Millipore) contributed to visualizing the signals.

### Statistical Analysis

Statistical analysis was conducted using SPSS 21.0 software. The experimental processes in the study were carried out in triplicate, and mean ± SD represents the results. The two-tail Student’s t-test was applied for assessing the difference between groups. Kaplan–Meier curve with log-rank test was utilized to compare the survival outcome. Pearson correlation analysis was performed to analyze the correlation between LINC00857, miR-150-5p, and E2F3. It was set that the difference was of statistical significance with P value < 0.05.

## Results

### LINC00857 Was Overexpressed in Pancreatic Cancer and Associated With Poor Clinical Outcomes

**Figure 1:**
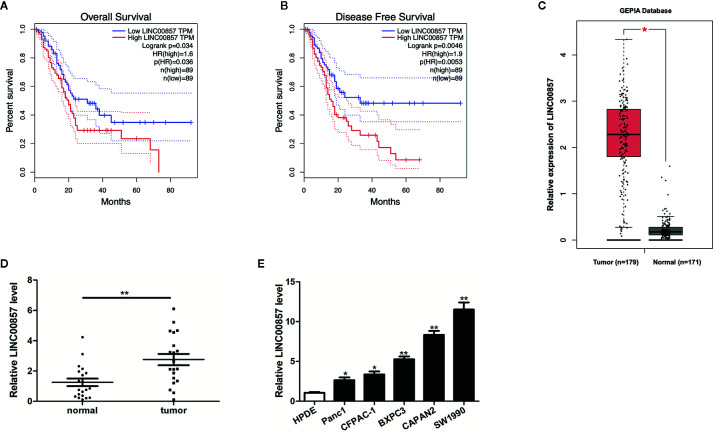
LINC00857 was overexpressed in pancreatic cancer and associated with poor clinical outcomes. **(A, B)** The association between LINC00857 expression and overall survival (OS) or disease-free survival (DFS) of patients with pancreatic cancer (PC) was analyzed *via* Gene Expression Profiling Interactive Analysis (GEPIA) database. OS: hazard ratio (HR)=1.6, P = 0.034; DFS: hazard ratio (HR)=1.9, P = 0.0046. **(C)** Expression of LINC00857 in PC samples was obtained in GEPIA database. **(D)** Relative expression of LINC00857 in PC and adjacent normal tissues (n = 20). **(E)** LINC00857 expression level in human pancreatic ductal epithelial (HPDE) cell and 5 PC cell lines. Data represent the mean ± SD. *P < 0.05, **P < 0.01. The experiments were independently repeated at least three times.

To identify lncRNAs involved in pancreatic cancer (PC) progression, the prognostic role of LINC00857 was analyzed *via* the GEPIA database. We classified the patients into high and low LINC00857 expressing groups based on the median expression value. The results showed that the overall survival rate (HR=1.6, p=0.034) and disease-free survival rate (HR=1.9, p=0.0046) of PC patients with high expression of LINC00857 were remarkably lower than those of low expression group (**Figures 1A, B**). In addition, the GEPIA database revealed that LINC00857 exhibited high expression in 179 PC samples compared with 171 non-tumor samples (**Figure 1C**). Then, we evaluated LINC00857 expression in 20 PC tissues and adjacent normal tissues by qRT-PCR. The results showed that LINC00857 was significantly upregulated in PC tissue samples compared with that in adjacent normal tissues (**Figure 1D**). Similarly, LINC00857 expression was remarkably higher in 5 PC cell lines than that in human pancreatic ductal epithelial (HPDE) cells, including Panc1 (p < 0.05), CFPAC-1 (p < 0.05), BXPC3 (p < 0.05), especially CAPAN2 (p < 0.01), SW1990 (p < 0.01) (**Figure 1E**). We focused on these two cells (CAPAN2 and SW1990) for our further study. To sum up, it can be judged that LINC00857 overexpression is a common phenomenon in PC and associated with poor clinical outcomes.

### m^6^A Modification Was Associated With the Upregulation of LINC00857 in PC Cells

**Figure 2:**
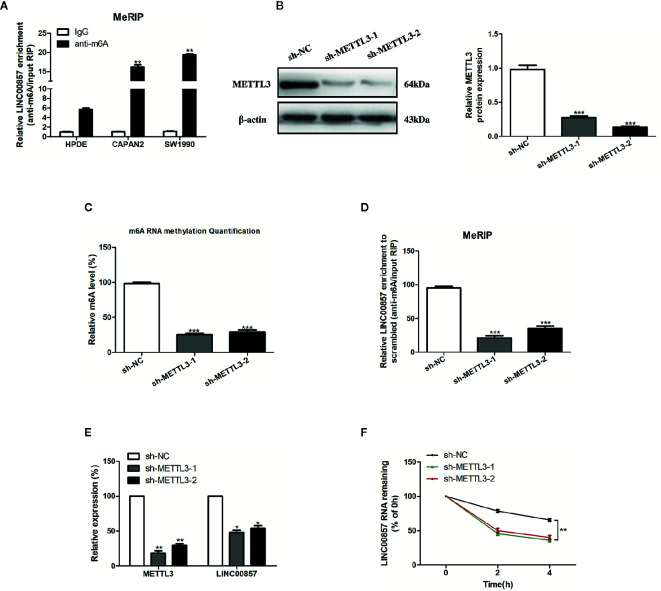
The m^6^A modification was enriched in LINC00857 and improved its transcripts stability. **(A)** m^6^A methylation level of LINC00857 in HPDE and pancreatic cancer (PC) cells (CAPAN2 and SW1990) were determined by MeRIP-qPCR. **(B)** The knockdown effect of sh-METTL3 was verified by Western blot (WB) analysis in SW1990 cells. **(C)** m^6^A methylation level in SW1990 cells after METTL3 depletion was analyzed by kit in SW1990 cells. **(D)** Changes in m^6^A-modified LINC00857 levels upon METTL3 slicing in SW1990 cells. **(E)** Transcript levels of METTL3 and LINC00857 in negative control and sh-METTL3 SW1990 cells. **(F)** Reduction of LINC00857 RNA stability in METTL3 knockdown SW1990 cells as compared to control. Cells were treated with actinomycin D and RNA was isolated at 0, 2, and 4 h. Data represent the mean ± SD. *P < 0.05, **P < 0.01, ***P < 0.001. The experiments were independently repeated at least three times.

Recently, the involvement of m^6^A modification in lncRNAs has been discovered in the research on tumor epigenetic regulation (18, 19). Thus, it is wondered if m^6^A modification is present in LINC00857. Based on the online bioinformatics database m6Avar (http://m6avar.renlab.org), we found four RRACU m^6^A sequence motifs in the exon region (at chr10:81978357, 81977798, 81977927, and 81978636). Next, we performed methylated RIP assay in HPDE and two PC cells (CAPAN2 and SW1990). According to the results, m^6^A was higher enriched within LINC00857 in CAPAN2 and SW1990 cells than that in HPDE (**Figure 2A**). METTL3 is a crucial m^6^A methyltransferase (20). We then performed shRNA-mediated silencing of METTL3 (**Figure 2B**) and found that downregulation of METTL3 resulted in the decreased m^6^A level of both total RNA and LINC00857 in SW1990 cells (**Figures 2C, D**). Then we explored whether m^6^A modification could affect LINC00857 RNA metabolism and found that knockdown of METTL3 led to lower expression of LINC00857 in SW1990 cells (**Figure 2E**). After new RNA synthesis was blocked with actinomycin D, we measured the loss of LINC00857. The results indicated that LINC00857 showed lower RNA stability after silencing of METTL3 in SW1990 cells (**Figure 2F**). It was suggested that the m^6^A level of LINC00857 was higher in PC cells, and its modification in LINC00857 improved transcripts stability.

### LINC00857 Promoted Proliferation and Inhibited the Apoptosis of PC Cells

**Figure 3:**
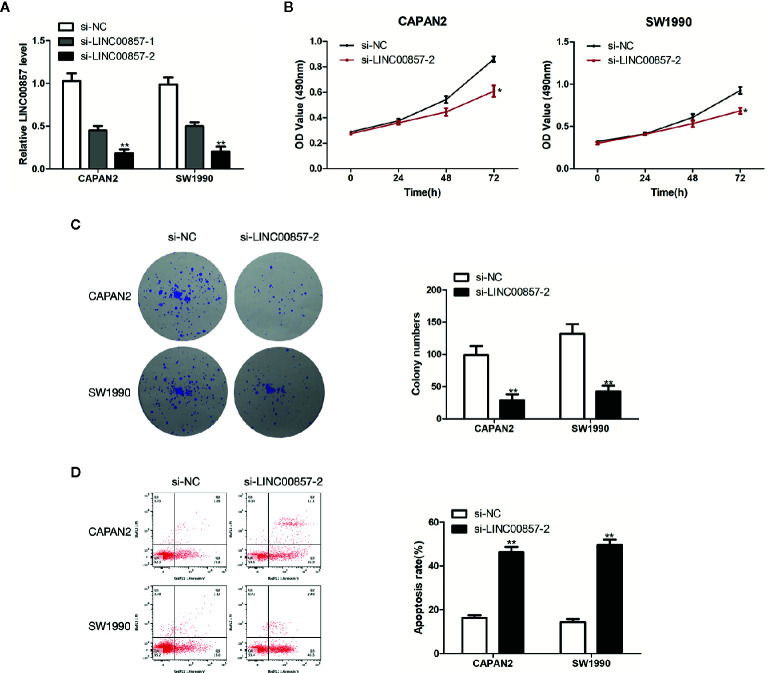
LINC00857 promoted proliferation and inhibited the apoptosis of pancreatic cancer (PC) cells. **(A)** Expression of LINC00857 in CAPAN2 and SW1990 cells transfected with si-LINC00857s or scrambled control. **(B)** The viability was detected by CCK-8 assay. **(C)** Representative images (left) and quantification (right) of the colony formation assay. **(D)** The apoptotic rate of cells was determined using flow cytometry. Data represent the mean ± SD. *P < 0.05, **P < 0.01. The experiments were independently repeated at least three times.

To assess the impact of LINC00857 on PC cells, we transfected CAPAN2 and SW1990 cells with two different siRNAs against LINC00857 (si-LINC00857-1 and si-LINC00857-2) and confirmed the transfection efficiency by qRT-PCR analysis. si-LINC00857-2 showed the most effective knockdown effect and was used for the subsequent experiments (**Figure 3A**). CCK-8 and colony formation assays revealed that the proliferation of PC cells transfected with si-LINC00857-2 was significantly decreased compared to scrambled group (**Figures 3B, C**). In addition, the apoptotic rate of PC cells transfected with si-LINC00857-2 was increased compared to scrambled group (**Figure 3D**). Taken together, these findings suggested that LINC00857 might have a potential to act as an oncogene in PC cells.

### LINC00857 Acted as a ceRNA and Competitively Absorbed miR-150-5p

**Figure 4:**
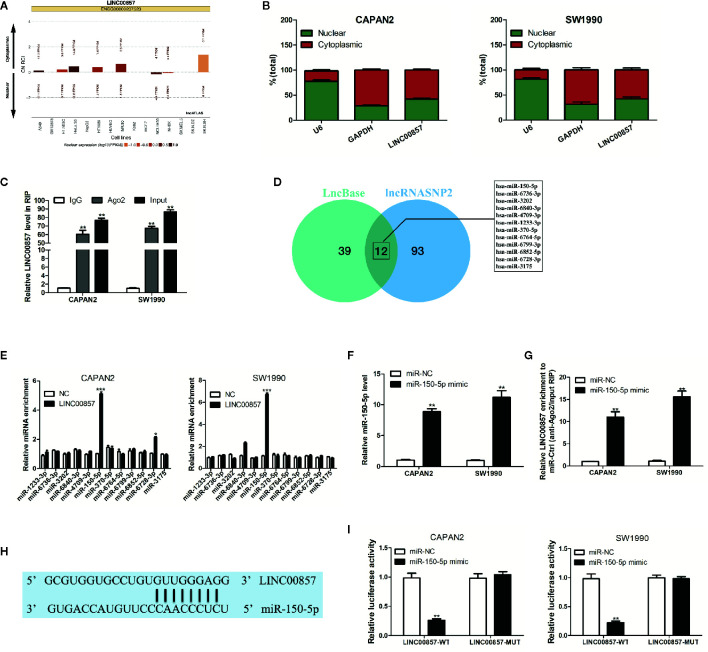
LINC00857 acted as a competing endogenous RNA (ceRNA) and competitively absorbed miR-150-5p. **(A)** LINC00857 was predicted to be located mainly in the cytoplasm using the bioinformatics tools in lncATLAS. **(B)** qRT-PCR analysis of LINC00857 expression in the nucleus and cytoplasm of CAPAN2 and SW1990 cells. U6 and GAPDH were used as endogenous controls. **(C)** RNA immunoprecipitation (RIP) assay for AGO2 was conducted to detect the level of endogenous LINC00857 in the AGO2 IP pellet. **(D)** A total of 12 miRNAs were predicted to harbor complementary sequences to LINC00857 according to LncBase and lncRNASNP2 databases. **(E)** RIP assays showed the enrichment of the predicted 12 miRNAs in pancreatic cancer (PC) cells with LINC00857 overexpression or NC. **(F)** MiR-150-5p expression was detected by qRT-PCR after transfection with miR-150-5p mimic. **(G)** Enrichment of LINC00857 in PC cells transfected with miR-150-5p mimics or miR-Ctrl. **(H)** Putative binding sequence between LINC00857 and miR-150-5p. **(I)** The luciferase activities in PC cells co-transfected with wild-type (WT) or mutant (Mut) LINC00857 plasmid together with miR-150-5p mimic or miR-Ctrl. Data represent the mean ± SD. *P < 0.05, **P < 0.01, ***P < 0.001. The experiments were independently repeated at least three times.

For understanding how LINC00857 exerts its function, lncATLAS (http://lncatlas.crg.eu/) was applied to predict the subcellular localization of LINC00857. The results indicated that LINC00857 was located mainly in the cytoplasm of a variety of cells (**Figure 4A**). qRT-PCR analysis in the nucleus and cytoplasm showed that LINC00857 was localized mainly in the cytoplasm in CAPAN2 and SW1990 cells (**Figure 4B**). RIP assay indicated that endogenous LINC00857 had a greater level of Ago2 IP pellet than control IgG IP pellet (**Figure 4C**). Thus, it was speculated that LINC00857 functioned as a ceRNA. Then we used LncBase (http://carolina.imis.athena-innovation.gr/diana_tools/web/index.php?r=lncbasev2%2Findex) and lncRNASNP2 (http://bioinfo.life.hust.edu.cn/lncRNASNP#!/) databases and discovered 12 miRNAs with a possibility to complement binding sequences (**Figure 4D**). According to RIP assays, relative to the negative control (NC) group, miR-150-5p was the most increased miRNA in the group with LINC00857-overexpression (**Figure 4E**). We transfected PC cells with miR-150-5p mimic and confirmed the transfection efficiency by qRT-PCR analysis (**Figure 4F**). In comparison with the miR-NC group, LINC00857 enrichment was much higher in the miR-150-5p mimic group (**Figure 4G**). It was indicated that LINC00857 and miR-150-5p were present in the same RNA-induced silencing complex. Moreover, a mutant sequence of LINC00857 was obtained that was incapable of binding miR-150-5p for the luciferase reporter assays (**Figure 4H**). According to **Figure 4I**, miR-150-5p mimic remarkably decreased the luciferase activity in the PC cells transfected with the wild-type (WT) LINC00857 sequence, while the luciferase activity showed no obvious alteration in cells transfected with the mutant (Mut) LINC00857. Thus, it can be judged that LINC00857 directly sponges miR-150-5p.

### miR-150-5p Was Down-Expressed in PC and Inhibited the Progression of PC Cells

**Figure 5:**
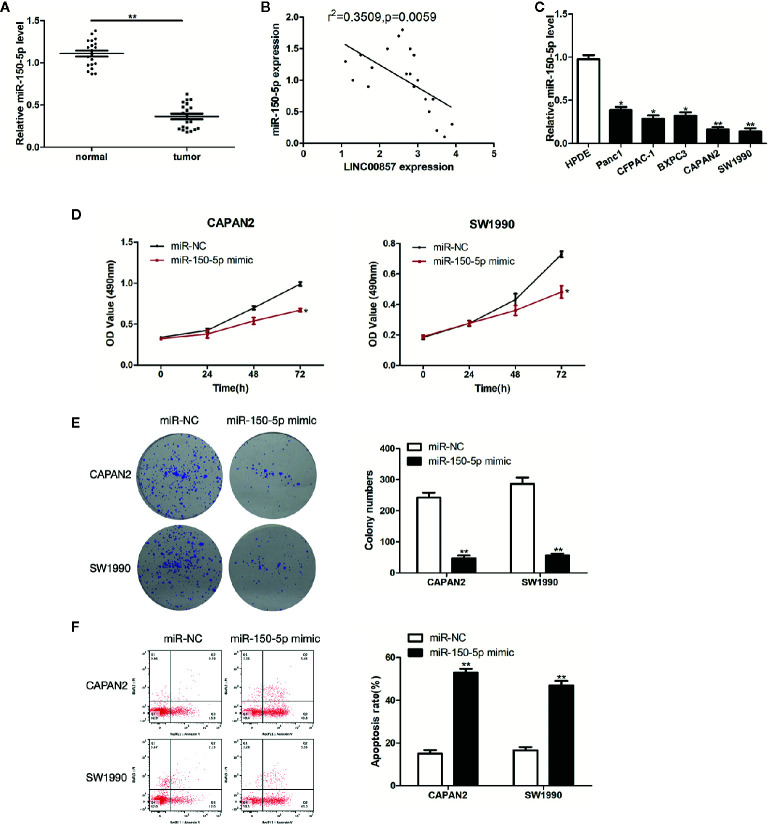
miR-150-5p was down-expressed in pancreatic cancer (PC) and inhibited the progression of PC cells. **(A)** Relative expression of miR-150-5p in PC and adjacent normal tissues (n = 20). **(B)** Pearson’s correlation scatter plot of LINC00857 and miR-150-5p in PC tissues. **(C)** MiR-150-5p expression level in HPDE and 5 PC cell lines. **(D)** The viability of cells transfected with miR-150-5p mimic or miR-Ctrl were detected by CCK-8 assay. **(E)** Representative images (left) and quantification (right) of the colony formation assay. **(F)** The apoptotic rate of cells was determined using flow cytometry. Data represent the mean ± SD. *P < 0.05, **P < 0.01. The experiments were independently repeated at least three times.

Next, the impact exerted by miR-150-5p on PC was explored. MiR-150-5p was down-expressed in PC tissues and cells (**Figures 5A, C**). Furthermore, Pearson correlation analysis revealed that miR-150-5p was moderately negative correlation with LINC00857 in PC tissues (**Figure 5B**). As revealed from the results of CCK-8 and colony formation assays, miR-150-5p overexpression restricted PC cells proliferation (**Figures 5D, E**). In addition, the apoptotic rate of PC cells transfected with miR-150-5p mimic was increased (**Figure 5F**). Given the mentioned data, miR-150-5p was down-expressed in PC and inhibited the progression of PC cells.

### LINC00857 Functioned *via* Negatively Regulating miR-150-5p Expression in PC Cells

**Figure 6:**
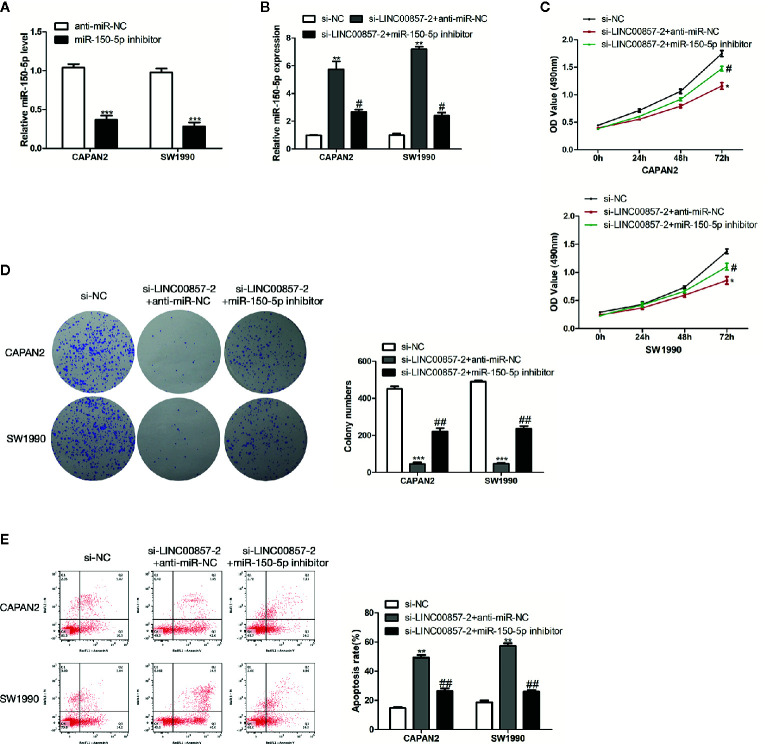
LINC00857 functioned *via* negatively regulating miR-150-5p expression in pancreatic cancer (PC) cells. **(A)** MiR-150-5p expression in PC cells was detected by qRT-PCR after transfection with miR-150-5p inhibitor. **(B)** MiR-150-5p expression in PC cells was detected by qRT-PCR after transfected with si-LINC00857-2 or co-transfected with si-LINC00857-2 and miR-150-5p inhibitor. **(C)** The viability was detected by CCK-8 assay. **(D)** Representative images (left) and quantification (right) of the colony formation assay. **(E)** The apoptotic rate of cells was determined using flow cytometry. Data represent the mean ± SD. *P < 0.05, **P < 0.01, ***P < 0.001, compared to control group; ^#^P < 0.05, ^##^P < 0.01, compared to si-LINC00857-2 group. The experiments were independently repeated at least three times.

miR-150-5p inhibitor underwent the transfection into the two PC cells (**Figure 6A**). MiR-150-5p expression was increased by si-LINC00857-2 and partially reversed through co-transfection with miR-150-5p inhibitor (**Figure 6B**). In the meantime, CCK-8 and colony formation assays showed that the cell proliferation inhibition mediated by si-LINC00857-2 could receive partial reversion by co-transfection with miR-150-5p inhibitor (**Figures 6C, D**), demonstrating that LINC00857 facilitated cell proliferation by restricting miR-150-5p expression. Additionally, flow cytometry assay showed that the apoptotic rate of PC cells was increased by si-LINC00857-2, and partially reversed through co-transfection with miR-150-5p inhibitor (**Figure 6E**). These results indicated that LINC00857 functioned *via* negatively regulating miR-150-5p expression in PC cells.

### miR-150-5p Directly Targeted E2F3

**Figure 7:**
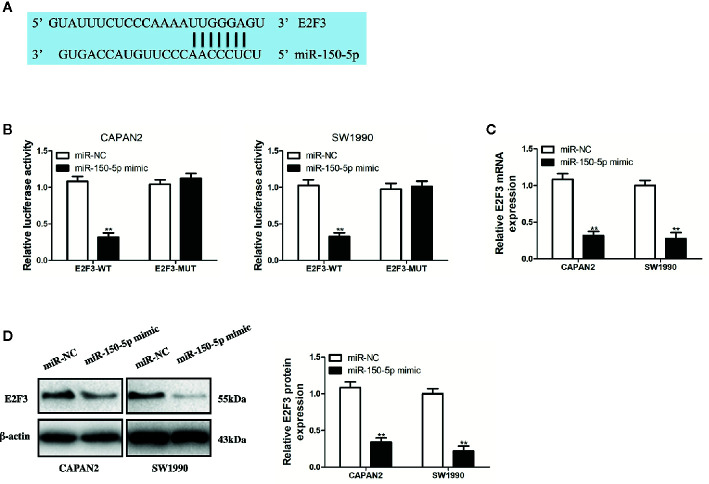
miR-150-5p directly targeted E2F3. **(A)** The predicted binding sites of miR-150-5p in the 3’-UTR of E2F3. **(B)** The luciferase activities in pancreatic cancer (PC) cells co-transfected with wild-type (WT) or mutant (Mut) E2F3 plasmid together with miR-150-5p mimic or miR-Ctrl. **(C, D)** qRT-PCR and Western blot (WB) analysis were used to measure the effect of miR-150-5p expression on the mRNA and protein expression of E2F3. Data represent the mean ± SD. **P < 0.01. The experiments were independently repeated at least three times.

For identifying the targets of miR-150-5p, bioinformatics analysis was conducted with TargetScan (http://www.targetscan.org/). A putative miR-150-5p binding site was identified in the 3’UTR of E2F3 (**Figure 7A**). miR-150-5p mimic suppressed the luciferase activity of E2F3-WT, but not for the activity of E2F3-MUT (**Figure 7B**). By detecting the mRNA and protein expression, it was discovered that the E2F3 expression in PC cells was inhibited by overexpressed miR-150-5p (**Figures 7C, D**). Thus, it was judged that miR-150-5p targeted E2F3.

### E2F3 Was Up-Expressed in PC and Responsible for LINC00857-Mediated Progression of PC Cells

**Figure 8:**
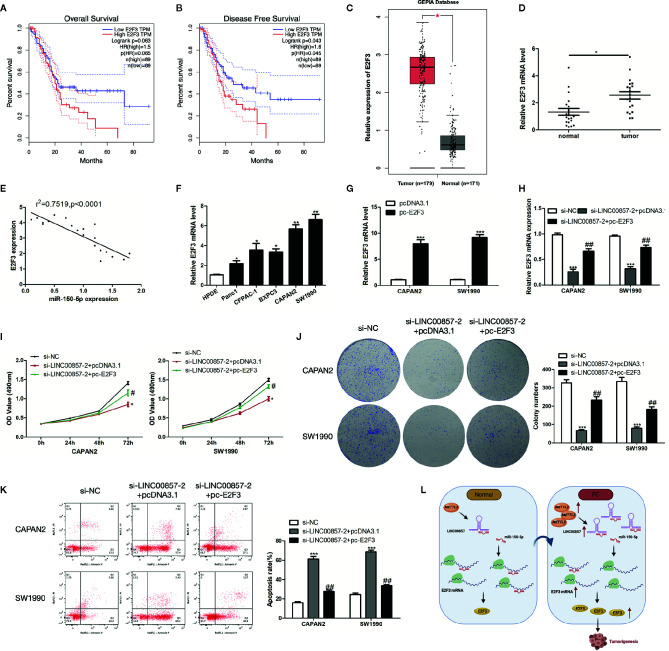
E2F3 was up-expressed in pancreatic cancer (PC) and responsible for LINC00857-mediated progression of PC cells. **(A, B)** The association between E2F3 expression and overall survival (OS) or disease-free survival (DFS) of patients with PC was analyzed *via* Gene Expression Profiling Interactive Analysis (GEPIA) database. OS: hazard ratio (HR)=1.5, P = 0.063; DFS: hazard ratio (HR)=1.6, P = 0.043. **(C)** Expression of E2F3 in PC samples was obtained in GEPIA database. **(D)** Relative expression of E2F3 in PC and adjacent normal tissues (n = 20). **(E)** Pearson’s correlation scatter plot of E2F3 and miR-150-5p in PC tissues. **(F)** E2F3 expression level in human pancreatic ductal epithelial (HPDE) and five PC cell lines. **(G)** E2F3 expression in PC cells was detected by qRT-PCR after transfection with pc-E2F3 or empty vector. **(H)** qRT-PCR was performed to detect E2F3 expression in PC cells after transfected with si-LINC00857-2 or co-transfected with si-LINC00857-2 and pc-E2F3. **(I)** The viability was detected by CCK-8 assay. **(J)** Representative images (left) and quantification (right) of the colony formation assay. **(K)** The apoptotic rate of cells was determined using flow cytometry. (**L**) Schematic diagram demonstrating the molecular mechanisms underlying LINC00857 in PC. Data represent the mean ± SD. *P < 0.05, **P < 0.01, ***P < 0.001, compared to control group; ^#^P < 0.05, ^##^P < 0.01, compared to si-LINC00857-2 group. The experiments were independently repeated at least three times.

To identify E2F3 involved in PC progression, the prognostic role of E2F3 was analyzed *via* the GEPIA database, which showed that the overall survival rate (HR=1.5, p=0.063) and disease-free survival rate (HR=1.6, p=0.043) of PC patients with high expression of E2F3 were remarkably lower than those of low expression group (**Figures 8A, B**). Besides, the GEPIA database displayed a significant upregulation of E2F3 in PC samples in contrast to the normal samples (**Figure 8C**). Then, we verified it with our specimens and found that E2F3 was up-expressed in PC tissues and cells (**Figures 8D, F**). Besides, E2F3 expression was found to exhibit an obvious negative correlation with miR-150-5p (**Figure 8E**). To determine the regulatory relationship between E2F3 and LINC00857, we transfected PC cells with E2F3 overexpression plasmid (pc-E2F3) and confirmed the transfection efficiency by qRT-PCR analysis (**Figure 8G**). It was demonstrated that si-LINC00857-2 inhibited the expression of E2F3, while pc-E2F3 could improve the expression (**Figure 8H**). CCK-8 and colony formation assays showed that the proliferation inhibition mediated by si-LINC00857-2 could be partially reversed by co-transfection with pc-E2F3 (**Figures 8I, J**), demonstrating that LINC00857 inhibited cell proliferation by regulating E2F3 expression. Moreover, flow cytometry indicated that the apoptotic rate was up-regulated by si-LINC00857-2 and partially reversed through co-transfection with pc-E2F3 (**Figure 8K**). The mentioned results suggested that E2F3 is responsible for LINC00857-mediated progression of PC cells. Taken together, our study revealed that LINC00857 exerted its function as a ceRNA through sponging miR-150-5p to regulate E2F3 expression, and therefore contributed to the progression of PC cells (**Figure 8L**).

## Discussion

Increasing evidence indicates that lncRNAs dysregulation plays important role in the tumorigenesis and progression of various kinds of cancers. Searching from GEPIA database, we found that high expression of LINC00857 was associated with poor prognosis in PC. Previously, LINC00857 has been reported to be highly expressed in other cancers. Here, we found that LINC00857 was upregulated and associated with worse clinical outcomes in PC. Then, we verified that LINC00857 was significantly upregulated in PC tissue samples and PC cell lines. Thus, we determined that LINC00857 is overexpression in PC and associated with poor clinical outcomes.

Recently, remarkable progress has been made in m^6^A modification for the regulation on the RNA life cycle at various stages (21). The dynamic and reversible modification of m^6^A installed and erased by “writers” capable of catalyzing the generation of m^6^A (such as METTL3 and METTL14), “erasers” that are selective in the removal of the m^6^A code (such as FTO and ALKBH5), and “readers” capable of decoding m^6^A methylation (such as YTH domain proteins and IGF2BP) (22). It was demonstrated that m^6^A alteration may have an impact on the targeted mRNA or miRNA and is related to the progression of various cancers (23–25). Nevertheless, the research into m^6^A modification in lncRNAs is limited. Recently, Zuo et al. proposed that m^6^A methylation was enriched within LINC00958 to affect its RNA stability in HCC cells (26). Herein, it was discovered that m^6^A methylation was increased within LINC00958 in PC cells. Additionally, METTL3 regulated the m^6^A modification in LINC00857, thus affecting its RNA stability. It was thus speculated that enhancement of LINC00857 in PC may be attributed to the m^6^A modification.

It has been increasingly evidenced that there is a novel and extensive interaction network involving ceRNAs (27, 28), where lncRNAs regulate miRNAs by binding their target sites on protein-coding mRNA molecules. For example, LINC00461 functions as a ceRNA to regulate ZEB2 expression by sponging miR-30a-5p to promote progression and malignancy in non-small cell lung cancer (29), SNHG15 functions as a ceRNA to promote KLF9-mediated proliferation by acting as a decoy for miR-141-3p in nasopharyngeal carcinoma (30), and DANCR promotes osteosarcoma migration and invasion by acting as a ceRNA that sponges miR-149 to regulate MSI2 expression (31). In this study, it was discovered that LINC00857 was located largely in the cytoplasm of PC cells, and functioned as a sponge for miR-150-5p.

MiR-150-5p is reported to be downregulated in different cancers and acts as a tumor suppressor miRNA (32, 33). Moreover, lncRNA PART1 is reported to function as a ceRNA to enhance CTNNB1 expression and activate Wnt/β-catenin pathway by competitively sponging miR-150-5p in colorectal cancer (34). In addition, miR-150-5p is reported to inhibit hepatoma cell migration and invasion by regulating MMP14 (35). Our findings revealed the importance of the association between LINC00857 and miR-150-5p in tumorigenesis. LINC00857 plays a carcinogenic role in PC by promoting cell proliferation, which can be partially rescued by overexpressed miR-150-5p.

As a ceRNA, the role of a lncRNA depends on the miRNA target. Using an online database, we predicted E2F3 as a potential target of miR-150-5p, which was affirmed by luciferase reporter assay. Furthermore, overexpression of miR-150-5p inhibited E2F3 mRNA and protein expression. E2F3, a member of the E2F family, was a crucial oncogene in several tumors (36). Previous studies have shown that E2F3 contributed to proliferation through regulating the cell cycle in NSCLC (37). miR-203a targeted E2F3 in gastric cancer to inhibit proliferation and colony formation (38). Additionally, miR-217 repressed growth and invasion of pancreatic cancer cells by regulating E2F3 (39). To identify E2F3 involved in PC progression, we used the GEPIA database, which showed that high expression of E2F3 was associated with well prognosis in PC. Then, we verified that E2F3 was up-expressed in PC tissues and cells. Herein, it was indicated that E2F3 was a target of miR-150-5p, promoting PC cell proliferation.

In conclusion, we identified LINC00857 as an oncogenic lncRNA in PC. Functional and mechanistic analyses revealed that LINC00857 promotes proliferation and inhibits the apoptosis of PC cells by acting as a ceRNA that sponges miR-150-5p, leading to enhanced E2F3 expression. Our study demonstrates that LINC00857 is significant to PC tumorigenesis, and highlights its value as a diagnostic indicator and suitable therapeutic target in PC.

## Data Availability Statement

The datasets presented in this study can be found in online repositories. The names of the repository/repositories and accession number(s) can be found in the article/**Supplementary Material**.

## Ethics Statement

The studies involving human participants were reviewed and approved by 2020-S460. The patients/participants provided their written informed consent to participate in this study.

## Author Contributions

HZ and XM conceived and designed the experiment. YD and SH performed most of the experiments. LN contributed the patient samples. YD analyzed the statistical analyses. XM wrote the manuscript. All authors revised the manuscript. All authors contributed to the article and approved the submitted version.

## Funding

This work was supported by the National Natural Science Foundation of China (82000614), the Natural Science Foundation of Hunan Province (2020JJ5876), the Science and Technology Plan Project of Changsha (kq2004146), the Hunan Provincial Science and Technology Plan Project (2019JJ80066), and the Scientific Research Project of Health and Family Planning Commission of Hunan Province of China (B20-17202).

## Conflict of Interest

The authors declare that the research was conducted in the absence of any commercial or financial relationships that could be construed as a potential conflict of interest.
