# Supplementary material for: m6A-Mediated Upregulation of LINC00857 Promotes Pancreatic Cancer Tumorigenesis by Regulating the miR-150-5p/E2F3 Axis
Source: Front Oncol. 2021 Feb 18;11:629947. doi: 10.3389/fonc.2021.629947 (PMC7930559; doi:10.3389/fonc.2021.629947)
Supplement: Supplementary file 1 [file Table_1.pdf]

Supplementary Table 1

	Sequences	
sh-METTL3-1	5′-GCCAAGGAACAATCCATTGTT-3′	
sh-METTL3-2	5′-GCTGCACTTCAGACGAATTAT-3′	
si-LINC00857-1	F: 5′-GGUAAGGGAAGGUGGAGAAUU-3′	R: 5′-UUCUCCACCUUCCCUUACCUU-3′
si-LINC00857-2	F: 5′-GGUAAGGGAAGGUGGAGAAUU-3′	R: 5′-UUGUUCACAGCACAUAGCCUU-3′
miR-150-5p mimic	5′-CTGGTACAGGCCTGGGGGACAG-3′	
miR-NC	5′-TTCTCCGAACGTGTCACGTAA-3′	
miR-150-5p inhibitor	5′-CTGTCCCCCAGGCCTGTACCAG-3′	
anti-miR-NC	5′-TTCTCCGAACGTGTCACGTAA-3′	
